# Metadata Correction: Impact of Educational Level on Study Attrition and Evaluation of Web-Based Computer-Tailored Interventions: Results From Seven Randomized Controlled Trials

**DOI:** 10.2196/jmir.5281

**Published:** 2015-11-10

**Authors:** Dominique A Reinwand, Rik Crutzen, Iman Elfeddali, Francine Schneider, Daniela Nadine Schulz, Eline Suzanne Smit, Nicola Esther Stanczyk, Huibert Tange, Viola Voncken-Brewster, Michel Jean Louis Walthouwer, Ciska Hoving, Hein de Vries

**Affiliations:** ^1^ CAPHRI Department of Health Promotion Maastricht University Maastricht Netherlands; ^2^ Jacobs Center for Lifelong Learning and Institutional Development Jacobs University Bremen Germany; ^3^ GGz Breburg Tilburg Netherlands; ^4^ Tranzo Department Tilburg University Tilburg Netherlands; ^5^ Amsterdam School of Communication Research (ASCoR) Department of Communication Science University of Amsterdam Amsterdam Netherlands; ^6^ CAPHRI Department of Family Medicine Maastricht University Maastricht Netherlands

The authors of the paper entitled “Impact of Educational Level on Study Attrition and Evaluation of Web-Based Computer-Tailored Interventions: Results From Seven Randomized Controlled Trials” [J Med Internet Res 2015;17(10):e228] inadvertently omitted Eline Suzanne Smit, PhD [CAPHRI, Department of Health Promotion, Maastricht University, Maastricht, Netherlands Amsterdam School of Communication Research (ASCoR), Department of Communication Science, University of Amsterdam, Amsterdam, Netherlands] from the list of authors during the copy-editing process. The author Eline Suzanne Smit was originally listed after Daniela Nadine Schulz in the list of authors. Additionally, a degree (PhD) has now been added for Hein de Vries. These errors have been corrected in the online version of the paper on the JMIR website on November 2, 2015, together with publishing this correction notice. Because these were made after submission to PubMed and other full-text repositories, the correction notice has been submitted to PubMed, and the original paper has been resubmitted to PubMed Central. The corrected metadata have also been resubmitted to CrossRef.

